# Engineering CRISPR/Cas13 System against RNA Viruses: From Diagnostics to Therapeutics

**DOI:** 10.3390/bioengineering9070291

**Published:** 2022-06-29

**Authors:** Yi Xue, Zhenzhen Chen, Wenxian Zhang, Jingjing Zhang

**Affiliations:** State Key Laboratory of Analytical Chemistry for Life Science, School of Chemistry and Chemical Engineering, Chemistry and Biomedicine Innovation Center (ChemBIC), Nanjing University, Nanjing 210023, China; mg20240117@smail.nju.edu.cn (Y.X.); ohguaiho@163.com (Z.C.); zhang.wenxian@foxmail.com (W.Z.)

**Keywords:** CRISPR, Cas13 system, RNA viruses, diagnostics, therapeutics

## Abstract

Over the past decades, RNA viruses have been threatened people’s health and led to global health emergencies. Significant progress has been made in diagnostic methods and antiviral therapeutics for combating RNA viruses. ELISA and RT-qPCR are reliable methods to detect RNA viruses, but they suffer from time-consuming procedures and limited sensitivities. Vaccines are effective to prevent virus infection and drugs are useful for antiviral treatment, while both need a relatively long research and development cycle. In recent years, CRISPR-based gene editing and modifying tools have been expanded rapidly. In particular, the CRISPR-Cas13 system stands out from the CRISPR-Cas family due to its accurate RNA-targeting ability, which makes it a promising tool for RNA virus diagnosis and therapy. Here, we review the current applications of the CRISPR-Cas13 system against RNA viruses, from diagnostics to therapeutics, and use some medically important RNA viruses such as SARS-CoV-2, dengue virus, and HIV-1 as examples to demonstrate the great potential of the CRISPR-Cas13 system.

## 1. Introduction

Throughout the history of human suffering and struggling against infectious diseases induced by viruses, the majority have been RNA viruses. This is exemplified by the outbreak of coronavirus disease in 2019 (COVID-19) induced by severe acute respiratory syndrome-related coronavirus 2 (SARS-CoV-2) [1,2,3,4]. As of 31 May 2022, COVID-19 had claimed more than six million lives, and nearly 530 million people have suffered from it (https://covid19.who.int (accessed on 31 May 2022)). In addition, many other RNA viruses threaten the worldwide public health and economic burden, such as dengue virus, which infects 96 million annually across more than 100 countries [5], and human immunodeficiency virus (HIV), with over 37 million people infected globally (https://www.hiv.gov/hiv-basics/overview/data-and-trends/global-statistics (accessed on 31 May 2022)) [6]. Due to the extremely high evolutionary rates of RNA viruses, it is trickier for humans to detect their infections than DNA viruses, which makes it too late to treat the related diseases. Consequently, to combat RNA viruses, effective methods for the diagnostics and therapeutics of RNA viruses are in urgent need [7,8].

The Clustered Regularly Interspaced Short Palindromic Repeats/Cas (CRISPR-Cas) systems, known as the bacterial immune systems against invading viruses, provide adaptative immunity to the hosts against invading nucleic acids [9]. Despite the useful DNA-targeting CRISPR-Cas9 system, the recently discovered RNA-targeting CRISPR-Cas13 system has shown its great potential in gene silencing without altering the whole genome. In vivo, the CRISPR-Cas13 system demonstrated a more efficient RNA knockdown property when compared to traditional RNAi technology [10]. Moreover, the CRISPR-Cas13 system was discovered to hold a collateral activity of cleaving RNA promiscuously in vitro [11]. According to the unique characteristics of the CRISPR-Cas13 system, researchers have recently harnessed the CRISPR-Cas13 system to combat RNA viruses [12].

In this review, we highlight the use of the CRISPR-Cas13 system for diagnosis and therapeutics of some representative and important RNA viruses such as SARS-CoV-2, dengue virus, and HIV-1. Several reviews have focused on the CRISPR-Cas systems’ emerging applications for infectious diseases diagnosis and antiviral therapy [13,14,15]. However, there is no review focusing on the rising star of the CRISPR-Cas13 system. Here, we would like to provide a perspective of the RNA-targeting system, the CRISPR-Cas13 system, to combat various RNA viruses, from diagnostics to therapeutics.

## 2. The CRISPR/Cas13 System

**Figure 1:**
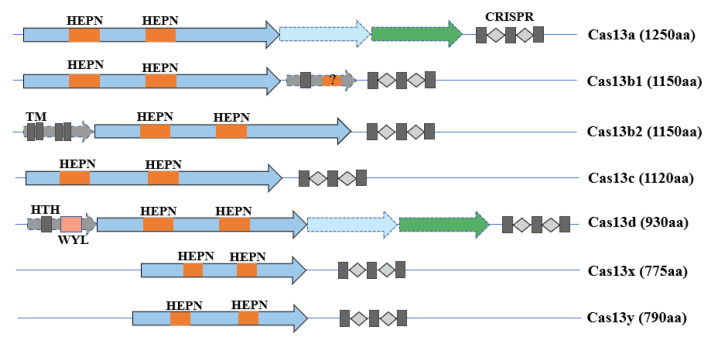
Constitutions of different Cas13 subtypes.

The CRISPR-Cas13 system, which is made up of dual components—a corresponding CRISPR RNA (crRNA) and a single Cas13 nuclease—belongs to the Class 2 Type VI CRISPR-Cas systems that contrast with Class 1 CRISPR-Cas systems, in which the nuclease complex consists of multiple subunits [16]. According to a phylogenic study [17], Cas13 proteins can be specifically classified into Cas13a, Cas13b (Cas13b1, Cas13b2), Cas13c, Cas13d, Cas13x, and Cas13y. They all have two higher eukaryotes and prokaryotes nucleotide-binding (HEPN) domains that are essential for RNA degradation (Figure 1) [18]. Mutations on HEPN domains would lead to a catalytically inactive protein that keeps its target RNA binding ability, which is an essential feature in RNA imaging, RNA tracking, and RNA modification [19]. Furthermore, just like the CRISPR-Cas12 system that possesses single-strand DNA collateral cleavage activity, the CRISPR-Cas13 system can nonspecifically degrade single-strand RNA (ssRNA) when activated by target RNA in vitro, a method that is widely used in diagnostics. However, the CRISPR-Cas13 systems cleave target RNA strands in a sequence-specific manner without the promiscuous cleavage ability in vivo, which is broadly applied in disease and antiviral therapies.

### 2.1. Cas13a

Cas13a, also named C2C2, was first introduced in 2015 by Zhang’s group using a computational pipeline strategy [16], and was identified in a subsequent experiment as a single-component programmable RNA-guided, RNA-targeting CRISPR effector [11]. To be programmed to degrade target ssRNA, the CRISPR/LshCas13a system needs to specify a 28 nt sequence on the crRNA and a 3′ non-G nucleotide protospacer-flanking sequence (PFS) motif adjacent to the protospacer target. In a lateral study, it was found that the CRISPR/LwaCas13a system did not require a PFS motif, which enriches Cas13a flexibility [20].

### 2.2. Cas13b

In addition, depending on a computational-sequence database-mining approach, Zhang et al. introduced Cas13b in 2017 [21], including two variants, Cas13b1 and Cas13b2, corresponding to the related accessory proteins Csx27 and Csx28. It is worth mentioning that Csx27 represses Cas13b activity, whereas Csx28 enhances it. In contrast to Cas13a, Cas13b has 1150 amino acids and needs a double-sided PFS, 5′ PFS of D (A, U or G), and 3′ PFS of NAN or NNA to maximize its targeting ability.

### 2.3. Cas13c

Cas13c was also discovered in 2017 when Zhang et al. sought to identify a more robust RNA-targeting CRISPR system [22]. Cas13c has a similar stereotypical locus and crRNA structure to Cas13a, with a size of 1120 amino acids. It was identified that Cas13c showed a weaker RNA interference ability than Cas13a and Cas13b in human embryonic kidney (HEK) 293FT cells.

### 2.4. Cas13d

Cas13d is a recently discovered type VI CRISPR-Cas variant with a smaller size of about 930 amino acids than Cas13a (1250aa) [10]. The crRNA of Cas13d comprises a 30 nt 5′ direct repeat followed by a variable 3′ spacer that ranges from 14–26 nt in length. The Cas13d-crRNA complex exerts rigorous sequence-specific RNA cleavage in vivo, which promises more distinct RNA interference in comparison to other systems, including RNAi, CRISPRi, and the other Cas13 members. Moreover, it has been shown that RNA target cleavage of CRISPR/Cas13d does not appear to depend on the PFS, which broadens its target-sequence choices.

### 2.5. Cas13X and Cas13Y

During the burst of Cas protein variants, Cas13X and Cas13Y were the latest to be found that held the smallest size of 775–803 amino acids [23], which could be truncated to 445 amino acids by structure-guided engineering, giving them a large advantage in delivery. Among the different CasX and CasY proteins, Cas13X.1 exhibits the highest knockdown efficiency and has no PFS bias.

## 3. Applications of CRISPR/Cas13 in RNA Virus Diagnostics

### 3.1. Conventional RNA Virus Diagnostics

**Figure 2:**
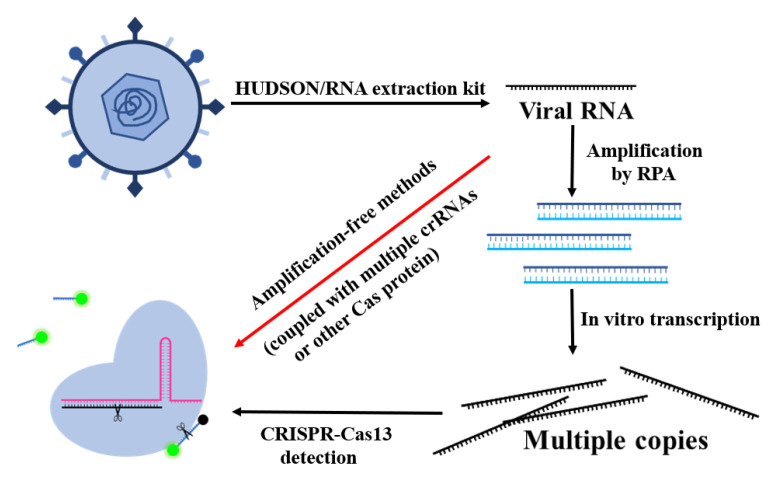
Strategies based on the CRISPR-Cas system to detect SARS-CoV-2.

**Figure 3:**
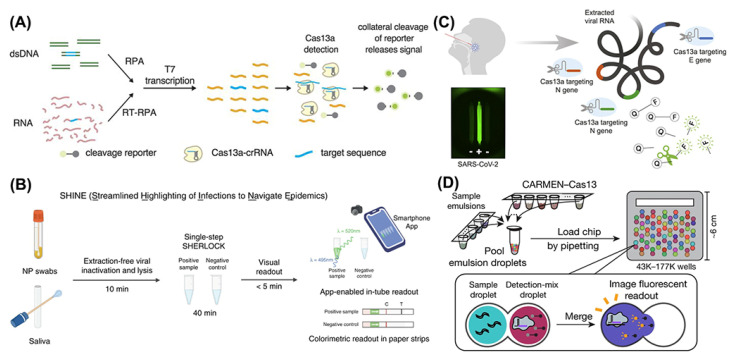
Schematic of different procedures of CRISPR-Cas13 based diagnostic methods: (**A**) schematic of SHERLOCK. Reprinted/adapted with permission from Ref. [31]. Copyright © 2022, American Association for the Advancement of Science. (**B**) schematic of SHINE. Reprinted/adapted with permission from Ref. [31]. Copyright © 2022, Springer Nature. (**C**) schematic of CRISPR-Cas13 system combined with mobile phone microscopy. Reprinted/adapted with permission from Ref. [34]. Copyright © 2022, Elsevier. (**D**) schematic of CARMEN. Reprinted/adapted with permission from Ref. [36]. Copyright © 2022, Springer Nature.

The conventional diagnostic tests for RNA-based viruses can be divided into two main types, the immunological and molecular methods [24]. The enzyme-linked immunosorbent assays (ELISA) can directly detect virus antigens or antibodies in the blood of infected people. It has been widely used in detections of the SARS-CoV-2 spike protein [25], dengue NS1 protein [26], and HIV p24 antigen [27]. The molecular assays are based on RNA detection with real-time quantitative reverse-transcription polymerase chain reaction (RT-qPCR) technology, which has become the gold standard for SARS-CoV-2 detection [28]. RNA viruses such as dengue virus and HIV-1 are also effectively detected with it [29,30]. Despite the wide use of ELISA and RT-qPCR, they are considered time-consuming and are heavily dependent on trained laboratory personnel. In addition, the demand for higher sensitivity and specificity led researchers to find some novel detection methods. CRISPR-Cas systems have always been outstanding gene-editing tools in the hands of biologists. However, as soon as Cpf1 was found to exhibit its collateral activity of cleaving DNA promiscuously in vitro, CRISPR-Cas systems also became popular among analysts. Compared with CRISPR-Cas9 and CRISPR-Cas12, the obvious advantage of CRISPR-Cas13 is its highly accurate RNA-targeting ability, which provides much convenience for direct RNA virus detection, especially in amplification-free methods based on CRISPR-Cas13 (Figure 2). CRISPR-Cas9 is rarely used in virus detection due to the lack of collateral cleavage ability, while CRISPR-Cas12 can only be activated by DNA, which is not suitable for RNA virus detection. Along with the report on Zhang’s “SHERLOCK” technology (Figure 3A) [31], the CRISPR-Cas13 system has demonstrated great promise in identifying RNA in a point-of-care (POC) testing format, without the requirement of expensive equipment and professionally skilled personnel. Additionally, the CRISPR/Cas13 system enables rapid, highly sensitive and selective, and cost-effective detection of various RNA samples.

### 3.2. CRISPR-Cas13-Based RNA Virus Diagnostics

#### 3.2.1. Applications of CRISPR-Cas13 in SARS-CoV-2 Detection

In 2017, Zhang’s group came up with a rapid, inexpensive, and sensitive nucleic acid detection method with CRISPR-Cas13a, termed Specific High-Sensitivity Enzymatic Reporter UnLOCKing (SHERLOCK) [31,32]. With a properly designed target RNA recognition, CRISPR-Cas13a could exhibit its promiscuous ribonuclease activity with a fluorescence reporter RNA. Single-molecule sensitivity has been achieved by the combinational amplification method of reverse transcriptase and recombinase polymerase amplification (RT-RPA) and T7 RNA polymerase transcription combined with the signal report of the collateral RNA cleavage ability of CRISPR-Cas13a. Based on that, they performed the clinical validation of a two-step assay for the detection of SARS-CoV-2 RNA. Through a comparison analysis of the limit of detection (LoD) of four selected gene regions of the SARS-CoV-2 genome map, it was found that the S gene gave the best performance in sensitivity for both lateral-flow strips and fluorescence tubes, which has been shown in 154 clinical samples with an 88% sensitivity on lateral-flow strips and 96% sensitivity on fluorescence signals with a detection limit of 42 RNA copies per reaction. Other common human coronaviruses have also been detected by SHERLOCK with no cross-reactivity results, showing that SHERLOCK holds a remarkable specificity. Not only that, it took SHERLOCK as little as 35 min to return a reliable detection result for strongly positive samples, which significantly reduced the test time compared to RT-qPCR by more than 120 min, excluding time for RNA extraction.

Focusing on the time needed for detection, Arizti-Sanz and colleagues developed an extraction-free, rapid, and sensitive diagnostic tool for SARS-CoV-2 RNA, termed Streamlined Highlighting of Infections to Navigate Epidemics (SHINE) [33]. Aiming to reduce the potential wasted time and unnecessary skilled operations in the two-step assay in a classical SHERLOCK test using extracted nucleic acids as input, they optimized a compatible condition for amplification and Cas13-based detection into a single-step SHERLOCK assay (Figure 3B). In addition, to avoid the extracting step usually required by a RNA extraction kit, they improved Heating Unextracted Diagnostic Samples to Obliterate Nucleases (HUDSON) from 30 to 10 min to lyse viral particles and inactivate the ribonucleases in bodily fluids. Combined with the faster HUDSON and single-step SHERLOCK, SHINE showed its great point-of-care potential with a 90% sensitivity and 100% specificity and a short test time of 50 min, RNA extraction time included, which should be emphasized.

However, to achieve high sensitivity, both SHERLOCK and SHINE used the preamplification process to lower the analytic limit of detection, which was considered time-consuming and to easily cause false-negative or false-positive results due to amplification errors. In an attempt to avoid that, Fozouni et al. developed an amplification-free CRISPR-Cas13a assay for direct detection of SARS-CoV-2 [34]. By activating more Cas13a per target RNA, they successfully detected SARS-CoV-2 at about 100 copies/µL in under 30 min of measurement time without an amplification step. What is worth mentioning is that the lack of an amplification process promised that the signal readout would directly correlate with the viral copy number, which was beneficial for quantitative tests and real-time monitoring of the course of patients’ infection degrees. When combined with a mobile phone camera as the fluorescence reader (Figure 3C), the Cas13a-crRNA assay could take the SARS-CoV-2 screening outside of laboratory settings, which enhanced its point-of-care applications.

In addition, as another target amplification-free method, the Fast Integrated Nuclease Detection in Tandem (FIND-IT) approach was developed by Liu and her colleagues [35]. By smartly assembling RNA-guided Cas13a and Csm6 with a specifically designed activator, multiple signal amplification was created to achieve a low sensitivity of about 30 molecules per µL of RNA in just 20 min. Generally, just like Cas13a, Csm6 can be triggered by a fixed short RNA sequence (cA4 or cA6) to activate multiple-turnover trans-cleavage of ssRNA. When taking A4-U6 as the trans-cleavage sequence of CRISPR-Cas13a upon target recognition, the release sequence of A4 would continue to activate Csm6 to produce an initial burst of fluorescence, which greatly accelerated SARS-CoV-2 detection.

Despite the creative methods based on the CRIPSR-Cas13 system that have been described above, a rapid, scalable, and easily deployable diagnostic tool for SARS-CoV-2 detection is still in great demand. As an extension of SHERLOCK, Ackerman et al. in 2020 described an attempt to enable scalable detection of many samples while simultaneously testing for many pathogens, SARS-CoV-2 included, named Combinatorial Arrayed Reactions for Multiplexed Evaluation of Nucleic acids (CARMEN) [36]. By combining the CRISPR-Cas13 system with the microwell-array system, CARMEN integrated high-throughput and rapid detection in a minimalized setting. Two categories of emulsified droplets were used in this platform—sample droplets that held target viruses RNA postamplification and unique dye-based color identifiers, and detection droplets that contained Cas13-crRNA and distinct fluorescent dye reporters with quenchers. The dye of each droplet was unrepeatable so that the reaction between a sample droplet and its matched detection droplet would be clearly identified by the two types of different fluorescent signal codes to determine the particular viral infection in the person from whom the sample came. Using CARMEN technology, more than 400 samples can be detected in parallel by just a single massive-capacity chip (Figure 3D).

However, CARMEN required custom-made chips and readout hardware, which hampered its application in a clinical setting. Lately, Nicole et al. upgraded CARMEN to microfluidic Combinatorial Arrayed Reactions for Multiplexed Evaluation of Nucleic Acids (mCARMEN) to scale up its diagnostic capability [37]. By replacing the custom microscope and chips with commercially available Fluidigm microfluidics and instrumentation, mCARMEN was able to identify SARS-CoV-2 variants such as Delta and Omicron in parallel in 300~550 patient specimens within 8 h and discriminate their unique mutations at a 5–10 times lower cost than next-generation sequencing.

In another study, Manning et al. presented a high-throughput SHERLOCK SARS-CoV-2 test that showed that all processes were automation-compatible in a single 384-well plate [38]. The workflow consisted of sample preparation using heat inactivation or RNA extraction, target amplification through loop-mediated isothermal amplification, and CRISPR-Cas13 with a fluorescence readout, which was finished within an hour. When combined with a high-throughput magnetic-bead-based purification, it provided a sensitivity down to 2 copies/µL for patient samples, or 10 copies/mL for direct patient samples, and the assay was 100% concordant with the RT-qPCR test. Moreover, with the help of a single liquid-handling robot, the high-throughput assay can process up to 5000 samples per day.

Despite Cas13a being used in RNA virus detection, Cas13d was also developed into a SARS-CoV-2 diagnostic assay, termed the Sensitive Enzymatic Nucleic-acid Sequence Reporter (SENSR) [39]. SENSR required an initial isothermal preamplification reaction combining RT-RPA producing a short dsDNA amplicon encompassing the Cas13d target site and containing a T7 promoter sequence. This is followed by an in vitro transcription (IVT)-coupled cleavage reaction that converts the dsDNA amplicons into ssRNA, recognizable by Cas13d for cleavage, resulting in collateral cleavage of a bystander ssRNA probe. Because the envelope (E) and nucleocapsid (N) genes within the SARS-CoV-2 genome are recommended in RT-qPCR assays by the WHO and CDC, Brogan et al. selected these genes as the targets of SENSR with a low LoD to be 100 copies/µL.

#### 3.2.2. Applications of CRISPR-Cas13 in Dengue Virus Detection

Having established a general detection method for RNA viruses based on SHERLOCK, Zhang’s group immediately set out to detect dengue viruses that were responsible for dengue hemorrhagic fever and dengue shock syndrome using this CRISPR-Cas13 system [40]. By testing multiple crRNAs with deliberately designed mismatches in the spacer sequences, SHERLOCK was able to recognize the corresponding dengue virus strains with a significantly high signal, which ensured robust sensitivity and specificity. In the following research, SHERLOCKv2 [40], they combined Cas13a and Cas13b with other CRISPR-associated protein nucleases such as Cas12 and Csm6 to realize multiple pathogen detections, including dengue virus, which were measured quantitatively as low as 2aM.They combined LwaCas13a, CcaCas13b, LbaCas13a, and PsmCas13b to detect different analytes with an independent reporter. To be specific, the four mentioned Cas proteins had their own RNA dinucleotide cleavage preference with reporters AU, UC, AC, and GA, which made them orthogonal in the same test tube. In particular, the cooperation of Cas13 and Csm6 enabled a 3.5-fold increase in signal sensitivity.

To optimize the unnecessary viral nucleic acid extraction step in both SHERLOCK and SHERLOCKv2, Myhrvold et al. also adopted HUDSON to pair with the CRISPR-Cas13a system to detect dengue virus [41]. With the use of heat and chemical reduction, viral particles could be lysed and RNases in bodily fluids would be inactivated. HUDSON-treated samples could be immediately used to carry out the SHERLOCK tests. Due to the convenient pretreatment method, dengue virus could be detected directly in patient samples within 2 h with a permitted attomolar level sensitivity.

In addition to that, a sensitive electrochemical detection method was used to detect dengue virus with the aid of CRISPR-Cas13a-assisted catalytic hairpin assembly [42]. Once the viral RNA activated the CRISPR-Cas13 system, promiscuous cleavage would release the blocker RNA that hybridized with swing RNA arms on the Au electrode. The arms would trigger the hairpin on the electrode surface to hybridize with the other free hairpin that was labeled with ferrocene to produce an amperometric signal for achieving signal amplification. With a detection limit of 0.78 fM, the proposed biosensor provides a simple and sensitive electrochemical method for dengue virus detection.

#### 3.2.3. Applications of CRISPR-Cas13 in HIV-1 Detection

HIV, the cause of AIDS, has an urgent demand for diagnosis as early as possible because infected patients have a relatively aggressive clinical course with the onset of immunodeficiency [43]. Therefore, applications of nucleic acid detection to HIV often require higher sensitivity. In their work on SHERLOCK, Zhang et al. verified that the detection limit based on CRISPR-LwaCas13a or CRISPR-PsmCas13b could be pushed beyond 2aM. In addition, in CARMEN [36], CRISPR-Cas13a has successfully identified HIV among various pathogens. In 2019, Fozouni et al. exploited the Cas13a enzyme complex with HIV crRNAs to detect and quantify the presence of HIV RNA in a sample specifically and sensitively [44]. After analyzing the HIV-1 RNA genome and avoiding the extensive secondary structure, they selected potential crRNAs and carried out tests on them. With the use of multiple good crRNAs, Cas13a/crRNA RNP complexes could detect target viral RNA down to 500 attomole, or 3.01 × 10^5^ copies/mL, which made the method sensitive and easily able to handle detection of HIV-1.

#### 3.2.4. Applications of CRISPR-Cas13 in Detection of Other RNA Viruses

In addition to SARS-CoV-2, dengue virus, and HIV, many other RNA viruses threatening human health could be detected in a rapid way by the CRISPR-Cas13 system. SHERLOCK was the first test used to detect Zika virus in clinical isolates as low as 2 × 10^3^ copies/mL. CARMEN has also been applied to differentiate 169 human-associated viruses, including RNA viruses such as Zika virus, hepatitis C virus, influenza A virus, etc. In 2019, Liu and his colleagues utilized the CRISPR-Cas13a nanomachine for avian influenza A (H7N9) on site detection by targeting the H7N9 HA gene [45]. Visual detection of porcine reproductive and respiratory syndrome viruses was realized by Chang et al. with the CRISPR-Cas13a-based lateral flow assay [46]. Ebola virus has also been detected in a rapid and microfluidic way based on the CRISPR-Cas13a system [47]. Moreover, Barnes et al. successfully detected and reported Ebola and Lassa virus cases in real time by combining HUDSON and SHERLOCK [48], as shown in Table 1.

## 4. Utilization of CRISPR/Cas13 in RNA Virus Therapeutics

### 4.1. Conventional RNA Virus Therapeutics

**Figure 4:**
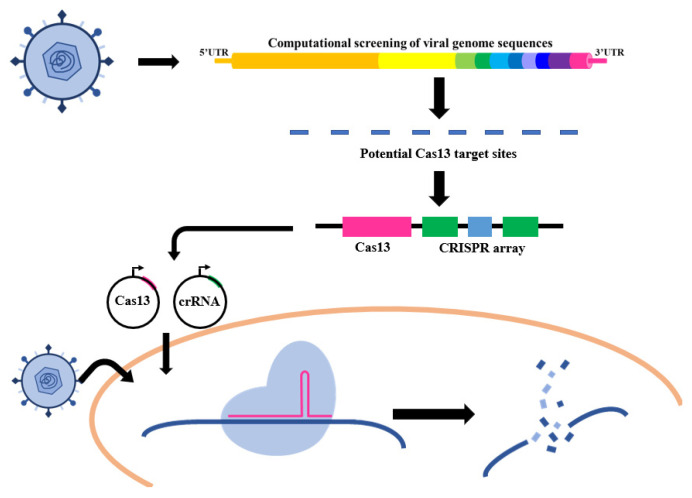
Strategies based on the CRISPR-Cas system for anti-RNA viruses.

Conventional RNA virus therapeutics place a focus on vaccination and antiviral drugs. As the primary strategy to prevent viral infection in hosts, vaccines have been widely used in combating viral diseases and have made a huge difference, but only a few viruses have FDA-approved vaccines. Even worse, the high evolutionary rates of RNA viruses often lead to vaccine failure. Following viral infections, small molecular antiviral drug treatments have become the only clinical therapy to resist viruses. However, the antiviral drug screenings rely highly on a biological or mechanistic understanding of virus infection, which takes much investigation time before a drug comes onto the market. Besides that, drug resistance and the performance of pathogen mutations should still be considered. Therefore, developing adaptable antiviral therapeutic platforms has always been kept in urgent demand due to the threat of RNA viruses. When applying CRISPR-Cas systems to RNA virus treatment, CRISPR-Cas9 and CRISPR-Cas12 are only appropriate for the certain RNA virus class that relies on its reverse-transcription ability to pass on the genetic material. However, CRISPR-Cas13 can be used in inhibiting any virus of which the genetic material is RNA. The CRISPR-Cas13 system that holds a specific RNA targeting ability offers the potential to combat RNA viruses in a “virus against virus” way (Figure 4).

### 4.2. CRISPR/Cas13-Based Anti-RNA Virus Therapeutics

#### 4.2.1. CRISPR-Cas13 in Anti-SARS-CoV-2 Therapy

In 2020, Abbott and his colleagues employed a prophylactic antiviral CRISPR in human cells (PAC-MAN) strategy to genetically intervene in SARS-CoV-2 infection [49]. Through a bioinformatic way of defining the conserved regions of the SARS-CoV-2 genome, they used a crRNA pool of six crRNAs able to effectively cleave the RNA sequences of the SARS-CoV-2 fragment with a 91% targeting efficiency, which expanded the CRISPR-Cas13 system applications from diagnosis to therapies. Specifically, they found the RdRP and N genes of SARS-CoV-2 that were essential for viral proliferation and packaging kept in an extremely highly conserved manner, which made them the perfect silencing targets of CRISPR-Cas13d. Research results verified that after post-transfection of Cas13d and crRNA pool plasmids, lung epithelial cells expressed the corresponding SARS-CoV-2 mRNA with a decline of 90%. Targeting and degrading the pathogen RNA by CRISPR-Cas13d have been identified to obtain a great SARS-CoV-2 inhibition effect.

By targeting the SARS-CoV-2 spike protein with CRISPR-Cas13a, Wang et al. also successfully eliminated viruses in human HepG2 and AT2 cells [50]. Given that the S gene of SARS-CoV-2 exhibited a relatively low mutation frequency, they used a bioinformatic method to identify a SARS-CoV-2-spike-specific segment and designed a crRNA library to target it. The qRT-PCR results showed that optimal crRNAs could achieve 99% of the knockdown efficiency. Furthermore, the catalytically inactive Cas13a was also investigated for its effect on spike protein expression in SARS-CoV-2. To the authors’ surprise, immunofluorescence results revealed a reduced S protein expression level by dCas13a-crRNA without altering the S RNA level in target cells. They attributed the effect to the interference of dCas13a-crRNA and S RNA, with no cleavage of S RNA but disordering of the translation process.

With the help of the smallest Cas protein, Cas13x, Xu et al. performed a bioinformatics analysis and selected crRNAs targeting RNA sites coding for RdRP and E proteins of SARS-CoV-2 to test Cas13X’s antiviral ability [23]. After cotransfection of HEK293T cells with the viral reporter gene and Cas13x/crRNAs, they observed that the viral RNA degrading efficiency could reach 70%, which promised Cas13x as an effective tool to combat SARS-CoV-2.

#### 4.2.2. CRISPR-Cas13 in Anti-DENV Therapy

Dengue virus, which is the most common arthropod-borne virus, has no specific therapy so far. As a single-stranded positive-sense RNA virus, dengue virus infection has been attempted to be treated using the CRISPR-Cas13 system. Li et al. discovered in 2019 that CRPSIR-Cas13a cleavage of the dengue virus NS3 gene could efficiently inhibit viral replication [51]. By screening of the dengue viral genome, they identified 10 vulnerable sites for CRISPR-Cas13a targeting. The designed crRNAs were formulated with the LwaCas13a protein to transfect DENV-2-infected Vero cells, which resulted in NS3-crRNA as the optimal crRNA. After three days post-transfection of the NS-crRNA-Cas13a complex and postinfection with dengue virus, the CRISPR-Cas13a system decreased viral RNA copy efficiently by 95%, providing a novel programmable anti-DENV strategy.

In another work, Singsuksawat et al. carried out a novel strategy by using Cas13b RNP with a short spacer that enhanced knockdown activity [52]. It has been proved that an 18–26 nt spacer would be suitable for RNA knockdown without compromising crRNA processing and multiplex targeting capability. Their screening analysis found 8681 crRNA to be the most efficient crRNA specific to DENV2-16681. Innovatively, they re-engineered a retrovirus to deliver Cas13b RNP to function in the cell immediately without genotoxicity. Applying these viruslike particles containing Cas13b RNP to primary human target cells, they achieved as small as a picomolar range of PspCas13b that resulted in a strong suppression of dengue infection.

#### 4.2.3. CRISPR-Cas13 in Anti-HIV-1 Therapy

In 2020, Yin et al. repurposed the CRISPR-Cas13 system to inhibit HIV-1 infection by targeting HIV-1 LTR, gag, tat, and rev regions [53]. By transfecting HEK293T cells infected by the HIV-1NL4-3-ΔE-YFP virus with plasmid DNA expressing LbuCas13a and crRNA, they found a 50% reduction in viral RNA by RT-qPCR and a 65–80% reduction by the reported YFP fluorescence. In addition, Western blotting of HIV-1 gag reflected a marked decrease caused by the CRISPR-Cas13a system. In addition, except for viral RNA, RNA from the transfected plasmid DNA and the integrated viral DNA were targeted and destroyed quickly as well. With strong inhibition of HIV-1 infection, it was thought that the CRISPR-Cas13a system would be a potential novel tool to combat HIV-1.

Besides Cas13a, the small Cas13d was used in HIV-1 treatment. Kulkarni’s group designed multiple crRNAs targeting the conserved regions of HIV-1 gag, pol, and cPPT genes [54]. Data showed the combination of multiple crRNAs with Cas13d reached a more than 90% inhibition efficiency in HIV replication, and they found that Cas13d functioned more effectively against HIV in the nucleus than in the cytoplasm, which corresponded with the results of Konnerman et al. showing that the nuclear localization signal increased Cas13d activity in mammalian cells. Because RNAi has been proved to be less effective in the nucleus, CRISPR-Cas13d with a better RNA-silencing performance would be a more powerful treatment tool.

#### 4.2.4. CRISPR-Cas13 in the Treatment of Diseases Caused by Other RNA Viruses

It should be noted that many other RNA virus infections could be treated by the CRIPR-Cas13 system as well. For example, Tng et al. found that a strong suppression of chikungunya virus RNA in infected insect cells was induced by Cas13b with suitable crRNAs [55]. Cui et al. adopted the CRISPR/Cas13b system for interference against the porcine reproductive and respiratory syndrome virus (pRRSV) RNA in eukaryotic cells, targeting its essential genes ORF5 and ORF7 [56]. The CRISPR-Cas13a system was also found to be effective at targeting the hepatitis C virus internal-ribosomal entry site for antiviral application [57]. In 2019, Freije et al. developed a Cas13-based platform called Cas13-assisted restriction of viral expression and readout (CARVER), and the tests showed its great antiviral performance in three cases: lymphocytic choriomeningitis virus (LCMV); influenza A virus (IAV); and vesicular stomatitis virus (VSV) [58]. In addition, the PAC-MAN and CRISPR-Cas13x systems were also used on the influenza A virus, as represented in Table 2.

## 5. Conclusions and Future Perspectives

RNA viruses have historically caused occasional epidemics and pandemics of highly contagious diseases. Mainly focusing on the three typical RNA viruses—SARS-CoV-2, dengue virus, and HIV-1—we showed the potential of the CRISPR/Cas13 system against RNA viruses from diagnostics to therapeutics. Given that the CRISPR-Cas13 system can promiscuously cleave ssRNA after target recognition in vitro, a variety of nucleic acid detection platforms have been developed. To improve the sensitivity of the CRISPR-Cas13-based detection method on RNA viruses, amplification techniques such as PCR, RPA, LAMP, transcription amplification, and so on have been elaborately incorporated into the detection system. Amplification-free detection platforms have also been explored using the combination of crRNAs or cooperation of Cas proteins to prevent wasted time and amplification errors. Moreover, rapid virus evolution and mutation has been considered as the major contribution factors to the misdiagnoses of RNA viruses, and serological antigen tests have been developed as a useful complement to improve the diagnostic accuracy [59,60,61]. In addition, by adopting different signal output modes such as lateral flow strips and mobile phone cameras as the fluorescence reader, the CRISPR-Cas13-based nucleic acid detection platforms might fulfill different detection requirements and be beneficial in point-of-care pathogen testing. Moreover, wearable materials with embedded CRISPR-Cas12 sensors for SARS-CoV-2 detection have been reported [62], and the widely used glucose meter has been transformed to detect SARS-CoV-2 [61]. An electrochemical biosensor based on an adaptor was successfully used for POC detection of SARS-CoV-2 combined with a smartphone [63]. Based on all these creative solutions aimed at POC tests, we do believe that an extensive collaboration of experts in chemistry, engineering, and computer science would further broaden the scope of CRISPR-Cas13-based diagnostic strategies, and potentially facilitate their translation into POC clinical applications to combat RNA viruses. Finally, high-throughput detection technologies based on the CRISPR-Cas13 system would aid in the restriction of the virus’ spread by large-scale detection of carrier individuals.

Nevertheless, not only diagnostic tools but also antiviral uses should be developed for the CRISPR-Cas13 system to help people combat RNA viruses. Always starting with the genome of a specific RNA virus, the potential crRNAs are designed to correspond to the most conserved gene, and then are combined with the Cas13 protein to perform their antiviral function. With high efficiency of target silencing, CRISPR-Cas13a, -Cas13b, and -Cas13d have been used in combating different RNA viruses, such as SARS-CoV-2, dengue virus, and HIV. However, it should be noted that the delivery methods and safety issues of the CRISPR-Cas13 system used in vivo are still immature, just like other CRISPR-Cas systems. There is great room for CRISPR-Cas13 system improvement in further applications. Upon use of CRISPR-Cas13a, C2C2 also was discovered, and researchers found its collateral activity could be directly observed biochemically in vitro and indirectly observed through growth suppression in bacteria. However, researchers have implemented the multiplexed leave-one-out and RNA-sequencing (RNA-seq) analyses, suggesting a lack of collateral RNA degradation in mammalian cells [22]. That makes CRISPR-Cas13 a reliable gene-editing therapeutic tool. In sum, the CRISPR-Cas13 system can be feasibly implemented for detection of RNA viruses and treatment of RNA virus infections, and may have the potential to show its distinct characteristics in this new age.

## Figures and Tables

**Table 1:** CRISPR-Cas13-based RNA virus diagnostics.

Virus	Gene Targets	LoD	Reference
SARS-CoV-2	S gene	42 copies per reaction	SHERLOCK [32]
	ORF1a	10 cp/µL using a fluorescent readout; 100cp/μL using the lateral-flow-based colorimetric readout	SHINE [33]
	N gene	30 copies/µL using a fluorescence plate reader; 100 cp/µL using mobile phone microscopy	[34]
	Total RNA	31 copies/µL	FINF-IT [35]
	Total RNA	Not mentioned	CARMEN [36]
	Total RNA	100 copies/µL	mCARMEN [37]
	N gene and ORF gene	5 copies/µL	[38]
	S gene and N gene	100 copies/µL	SENSR [39]
Dengue virus	Total RNA	2 aM	[40]
	Total RNA Total RNA	1 copy/µL 0.78 fM	[41] [42]
HIV-1	Not mentioned	Not mentioned	[44]
H7N9	HA gene	1 fM	[45]
PRRSV	M gene	172 copies/µL	[46]
Ebola virus	Total RNA	20 pfu/mL	[47]

**Table 2:** CRISPR-Cas13-based RNA virus diagnostics.

Virus	Cas	Gene Targets	Knockdown Efficiency	Reference
SARS-CoV-2	Cas13d	RdRP and N gene regions	90%	[49]
	Cas13a	S gene	99%	[50]
	Cas13X	RdRP and E gene	70%	[23]
Dengue virus	Cas13a	NS3 gene	95%	[51]
	Cas13b	NS5 gene	90%	[52]
HIV-1	Cas13a	LTR, gag, tat, and rev regions	50~80%	[53]
	Cas13d	Gag, pol, prot, int, cPPT, and CTS regions	90%	[54]
CHIKV	Cas13b	nsP2 gene	35~50%	[55]
PRRSV	Cas13b	ORF5 and ORF7 genes	55~70%	[56]
HCV	Cas13a	IRES	70~84%	[57]
LCMV	Cas13a	L and S segments	83.33%	[58]
IAV	Cas13b	mRNA and the complementary viral RNA	>85%	[58]
VSV	Cas13b	Single linear segment	>85%	[58]

## Data Availability

Not applicable.
